# Health beliefs, illness perceptions and determinants of breast screening uptake in Malta: a cross-sectional survey

**DOI:** 10.1186/s12889-017-4324-6

**Published:** 2017-05-08

**Authors:** Danika Marmarà, Vincent Marmarà, Gill Hubbard

**Affiliations:** 1 Faculty of Health Sciences, University of Stirling, Room E9, Pathfoot, Stirling, FK9 4LA Scotland, UK; 2 Cancer Care Pathways Directorate, Sir Anthony Mamo Oncology Centre, Level -1, Dun Karm Street, Msida, MSD 2090 Malta; 3 Department of Mathematics, University of Stirling, Stirling, FK94LA Scotland, UK; 4 Department of Management, University of Malta, Msida, Malta

**Keywords:** Breast cancer screening, Mammography, Uptake, Factors, Health belief model, Common-sense model

## Abstract

**Background:**

Women’s beliefs and representations of breast cancer (BC) and breast screening (BS) are salient predictors for BS practices. This study utilized the health belief model (HBM) and common-sense model (CSM) of illness self-regulation to explore factors associated with BS uptake in Malta and subsequently, to identify the most important predictors to first screening uptake.

**Methods:**

This cross-sectional survey enrolled Maltese women (*n =* 404) ages 50 to 60 at the time of their first screening invitation, invited to the National Breast Screening Programme by stratified random sampling, with no personal history of BC. Participants responded to a 121-item questionnaire by telephone between June–September 2015. Data were analyzed using descriptive statistics, chi-square tests and logistic regression.

**Results:**

There is high awareness of BC signs and symptoms among Maltese women (>80% agreement for 7 out of 8 signs), but wide variation about causation (e.g., germ or virus: 38.6% ‘agree’, 30.7% ‘disagree’). ‘Fear’ was the key reason for non-attendance to first invitation (41%, *n* = 66) and was statistically significant across all subscale items (*p* < 0.05). Most items within HBM constructs (perceived barriers; cues to action; self-efficacy) were significantly associated with first invitation to the National Breast Screening Programme, such as fear of result (χ2 = 12.0, *p* = 0.017) and life problems were considered greater than getting mammography (χ2 = 38.8, *p* = 0.000). Items within CSM constructs of Illness Representation (BC causes; cyclical cancer timeline; consequences) were also significantly associated, such as BC was considered to be life-changing (χ2 = 18.0, *p* = 0.000) with serious financial consequences (χ2 = 13.3, *p* = 0.004). There were no significant associations for socio-demographic or health status variables with uptake, except for family income (χ2 = 9.7, *p* = 0.047). Logistic regression analyses showed that HBM constructs, in particular perceived barriers, were the strongest predictors of non-attendance to first invitation throughout the analyses (*p* < 0.05). However, the inclusion of illness representation dimensions improved the model accuracy to predict non-attendance when compared to HBM alone (65% vs 38.8%).

**Conclusions:**

Interventions should be based on theory including HBM and CSM constructs, and should target first BS uptake and specific barriers to reduce disparities and increase BS uptake in Malta.

**Electronic supplementary material:**

The online version of this article (doi:10.1186/s12889-017-4324-6) contains supplementary material, which is available to authorized users.

## Background

Breast cancer (BC) is the most prevalent cancer in Europe [1], accounting for 28.8% of all female cancer incidences [2] with 425,000 new cases diagnosed yearly [3]. BC accounts for 21% of all female cancer incidences in Malta with an average of 280 women diagnosed each year, over the last decade [4].

Early detection of BC reduces morbidity and mortality, resulting in more effective treatment regimens and better survival rates [5]. Such mortality reductions are largely dependent on interventions, such as breast self-examination, clinical breast examination and screening [6]. Despite evidence that breast screening (BS) decreases BC mortality rates by 25–30% [7–9], BC screening rates remain suboptimal in many European countries [10, 11]. Although European Guidelines for Quality Assurance in BC [12] promote an acceptable target screening rate of at least 70%, and ideally 75% of eligible women [13], less than 60% of Maltese women accepted their first screening invitation [14] from a national breast screening programme, introduced in 2009 for women aged 50–60 years [13]. Since its establishment in Malta, the Maltese Breast Screening Programme (MBSP) routinely invites women free-of-charge by letter every three years and has expanded its cohort in its second screening round to include women aged 61–66 years.

Reasons for non-attendance are well documented and multifactorial [15, 16]. The extant literature suggests that a number of factors influence BS uptake, namely: (1) health beliefs [17–19], (2) illness representations [17, 20, 21], (3) knowledge of BC signs and symptoms, its causes and consequences, and recommended BS practices [22, 23], (4) socio-demographic factors [22, 24, 25], and (5) health status (medical factors) [17, 25–27].

### Theoretical framework: *The Health Belief Model and the Common-Sense Model of Illness Representation*

The Health Belief Model (HBM) was selected as one of the theoretical models for the current study, as it is widely used to identify associated variables with mammography and guides the prediction of screening behaviours [17, 18]. The HBM consists of six constructs: perceived susceptibility, perceived severity, perceived benefits, perceived barriers, cues to action, and self-efficacy [28, 29]. It proposes that the following factors play an important role in an individual’s perception about BS, such that women are more likely to perform BS if: a) they feel susceptible (vulnerable) to BC or the risks of contracting the disease (perceived susceptibility), b) believe in the seriousness of BC and its consequences for the individual (perceived severity), c) perceive more benefits than barriers from undergoing mammography, d) have higher confidence for obtaining a mammogram, and e) if a cue to action is present [28].

HBM, however, only explains some variation in BS behaviour [29], which is why the Common-Sense Model (CSM) of self-regulation [or Self-Regulation Model (SRM)], developed by Leventhal and colleagues in 1980s, has been used to consider the cognitive and emotional representations of an illness [17]. This study was also informed by the CSM to understand how individual symptoms and emotions influence one’s perception of BC, such as its likely impact upon physical and psychosocial functioning, and guide subsequent coping behaviour. Originally, illness representations comprised five components: *identity*, *cause*, *timeline*, *consequences,* and *cure/control* [17, 30]. These dimensions were further differentiated to include a further four dimensions: timeline cyclical; personal and treatment control; illness coherence and emotional representations [31].

Although screening behaviours can be predicted by knowledge, health beliefs and illness perceptions [17, 32], only a small body of research has jointly explored the latter to understand BS behaviour [17, 33], thereby limiting opportunities to examine if certain cognitions explain most of the variation in BS uptake. Furthermore, factors influencing uptake to a first BS invitation may differ to subsequent invitations, particularly since previous experience of BS is associated with future uptake [34]. Finally, the determinants of BS behaviour have not been studied in the Maltese population although determinants may not be comparable across different countries [35].

The primary aims of the study were:

1. To describe Maltese women’s knowledge, health beliefs and illness perceptions about breast cancer and screening;
2. To identify the main reasons related to non-attendance at the MBSP;
3. To determine if health beliefs, illness perceptions, knowledge, socio-demographic factors and health status are associated with uptake to first invitation at the MBSP;
4. To determine the significant predictors to first breast screening uptake.

‘Strengthening the Reporting of Observational Studies in Epidemiology’ (STROBE) guidelines [36] [see Additional file 1], have been used to present the study findings in this article. This is the first study of its kind in Malta; the findings could be used to inform future strategies and interventions to improve uptake in Malta, which as already highlighted, was sub-optimal for first round screening [14]. We hypothesized that there would be significant associations between health beliefs and illness perceptions, knowledge, socio-demographic factors, health status, and BS uptake.

## Methods

### Study design

A cross-sectional survey of women’s uptake of first invitation to the MBSP using validated tools to measure the influence of health beliefs and illness representations and using further questionnaires to measure knowledge of BS practices, socio-demographic and health status administered by telephone.

### Setting

The study was carried out in Malta between June 2015 and September 2015. Since there is only one Breast Screening centre (no mobile units), located in Malta’s capital city, Valletta, all data was generated from one computerized screening database and women were contacted from the centre.

### Participants

**Fig. 1:**
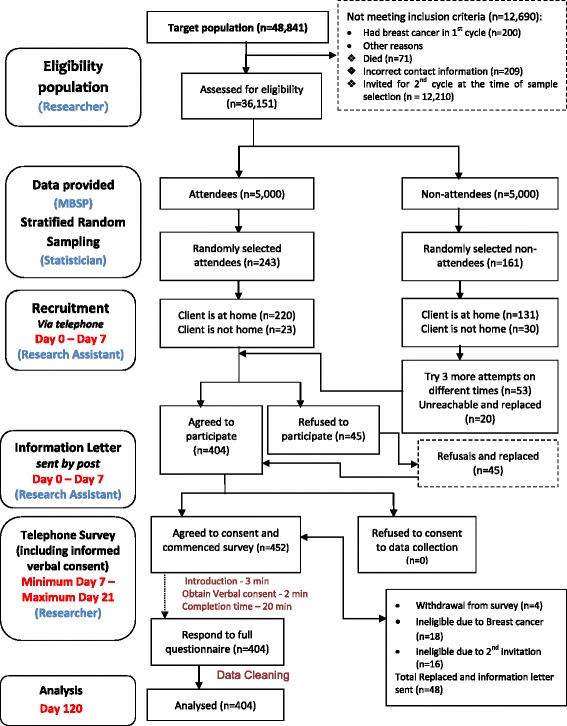
Participant pathway and sampling flowchart (based on STROBE guidelines)

The inclusion criteria were: women aged 50–60 at the time of their first screening invitation, residents in Malta or Gozo with a valid identity card number, able to communicate in English and Maltese, and with no severe co-morbidities. Women were excluded if they had ever been diagnosed with BC (*n* = 200), if they were invited to the second screening cycle (*n* = 12,210), if registered as deceased at the time of the sample selection (*n* = 71) and if incorrect information existed at the MBSP (*n* = 209) (Fig. 1).

### Sample size and sampling technique

In order to ensure that the study sample was nationally representative of the screening population and to decrease the margin of error in the estimation, women were selected by a stratified random sampling technique, employed by strata i.e. district (geographical distribution), age and attendance/non-attendance to the first BS invitation. According to the MBSP, the target population was estimated to be 48,841 women who were invited during the first screening cycle [37]. Following the exclusion of subjects (numbers in parentheses) in the sampling flowchart (Fig. 1), the eligible population was calculated to be equal to 36,151 women. A sample size of 404 women was determined using a 95% confidence level and a 5% confidence interval. In order to obtain this sample size, the following number of attendees and non-attendees were randomly selected as follows: *n* = 243 attendees [women’s reasons for attendance may provide a better insight to why people do not turn up for BS], and *n* = 161 non-attendees [this is representative from the actual population as 58.7% of those invited accepted their first invitation] [14]. Forty-five women refused to participate in this study (due to two reported personal reasons, i.e. lack of time due to work and family; fearful to speak about the topic under investigation). All 45 women were free to provide their own reason for non-participation. Content analysis of open-ended comments on reasons for refusal was employed, and later categorised and classified as being one of the above two reasons. Women’s comments were typical reasons for refusals in similar studies [38]. Due to the women’s refused participation, 449 women were eventually contacted in order to reach the necessary quota for each strata (with 90% response rate). Hence, the required total sample of 404 women was collected. Another 48 women were replaced during data collection since they were found to be ineligible during the telephone survey. All replacements were carried out in a way so as not to lose any of the sample representativeness of the population. Hence, replacements were selected with the same demographics of the non-respondent.

### Data collection

The participant recruitment pathway is presented in Fig. 1. Participants were recruited by telephone, by a trained research assistant who requested initial verbal consent. If the client agreed to participate, a brief explanation of the study was provided by telephone. Thereafter, a written information letter was posted to women on that same day. Hence, women received pre-notification letters to further inform the participant about the study’s aims, objectives and purpose, thus allowing the participant adequate time to read the information letter before further contact. Those who refused participation were deemed to have refused consent and were not contacted further. Scheduled appointments were set at women’s most convenient date and time (in around 7 days from first phone call) so that participants would not to be caught ‘off-guard’ when contacted by telephone, and also so that the researcher could conduct a telephone survey which was the chosen, feasible method for this study. Telephone surveys have also been utilised successfully in the extant literature [39, 40]. In cases of non-response, three call-backs were performed on different occasions, following which the researcher moved on to contact other participants.

Subjects were provided with information regarding the specific study aim, content and estimate time to respond to the survey, and that no incentive would be provided. Respondents were assured that all the collected information would be processed anonymously and confidentially. They were also informed that they could refuse to answer any question or decline participation at any point. For those participants who affirmed they were willing to respond, verbal informed consent was obtained by telephone through the use of standard procedures and guidelines [41]. Verbal consent is common practice when conducting survey interviews or interventions by telephone [39, 40, 42] and was chosen because it facilitates comprehension of study objectives and questionnaire items, and reduces the unnecessary burden entailed in a written consent form [39].

Participant recruitment by the research assistant was done manually, using paper format to record verbal consent by ticking Yes/No and to schedule appointments for the participants and the primary investigator (DM). The telephone survey was carried out by a single researcher (DM) and data entry was carried out (DM) through the use of computer-assisted technology through an online study tool (the SurveyMonkey program). Subsequently, the data were downloaded by the primary investigator (DM) from the same program. Minor formatting adjustments were made to the raw aggregate data in Microsoft Excel, and then the data were exported into the Statistical Package for the Social Sciences (SPSS). This method of handling data significantly decreased human error in the data entry process. This procedure of data storage and handling was secure, ensuring confidentiality of information provided by participants.

### Measures

The questionnaire was initially developed using previously validated questionnaires (CHBMS-MS and IPQ-R) [31, 43]. The CHBMS-MS and IPQ-R scales were used after securing written permission from the authors and were translated and adapted into the Maltese language and tested for validity in a pilot study involving 15 Maltese women [more information is available from the authors]. Our findings show overall positive correlation of the total inter-item correlation (CHBMS-MS: 0.87, IPQ-R: 0.85) (*p˂0.001* respectively), high Cronbach’s alpha (CHBMS-MS: 0.93, IPQ-R: 0.92), overall acceptable internal consistency (CHBMS-MS: 0.69–0.83, IPQ-R: 0.75–0.93), and acceptable test-retest reliability correlations: CHBMS-MS (Maltese: 0.62–0.76, English: 0.61–0.84); IPQ-R (Maltese: 0.63–0.82, English: 0.61–0.91) (*p˂0.001* respectively). Hence, this version of the instrument was used in this cross-sectional study.

The 121-item questionnaire is composed of four sections:

1. 11 subscales for socio-demographic and health status (20 items) related to age, residing district, education, employment, marital status, family income, car ownership/driving, illness/disability, having a GP, breast condition, family history of BC or other cancer. Response options were “yes”, “no” or a series of tick boxes. Open questions were asked when it was believed to be important that women could provide further detail, for example, type of illness, breast condition or cancer site.
2. 4 subscales for lifetime BS practices (17 items) that were clustered in 4 subscales: lifetime mammography use (4 items), attendance/non-attendance to first round screening (8 items), re-attendance/intention (4 items), knowledge about recommended screening frequency (1 item). Most of the response options were mostly designed to elicit “yes”, “no” or “unsure” answers. Closed questions allowed women to respond to a series of tick boxes.
3. 5 subscales for health beliefs (36 items) that were clustered into: perceived susceptibility (3 items), perceived benefits (6 items), perceived barriers (13 items), cues to action (7 items) and self-efficacy (7 items). All items had 5 response options ranging from: 1 = ‘strongly disagree’ to 5 = ‘strongly agree’. Reverse scoring (r) was performed for only one item ‘There is no possibility of getting breast cancer’ so that higher values would indicate greater possibility.
4. 7 subscales for illness perceptions (48 items) that were clustered into: breast cancer identity (8 items), causal scale (18 items), cancer timeline: acute/chronic (2 items), cyclical (1 item), consequences (8 items), curability/controllability (personal control - 3 items; treatment control - 3 items), illness coherence (2 items), and emotional representations (3 items). All items had 5 response options ranging from: 1 = ‘strongly disagree’ to 5 = ‘strongly agree’.

### Ethical considerations

Ethics approval was obtained from the School Research Ethics Committee at the University of Stirling (SREC14/15-Paper No.18v4) and by the Maltese Health Ethics Committee (HEC 02/2015). After securing written permission from the Chief Executive Officer, data were obtained from the MBSP and was computer generated from the local screening register.

### Variable definitions

A first invitation was defined as the first (initial) time a woman is invited to the MBSP and either attends or does not attend for the screening mammogram. Modifying factors include socio-demographic and health status variables (some of which were confirmed from women’s health records from the screening database), and structural variables such as knowledge of screening frequency and of the disease. These variables were collected from the survey administered retrospectively from the time of the first screening invitation.

### Data analysis

Data entry and statistical analysis were performed using SPSS® version 21.0 under direct instruction and guidance of an expert statistician. Descriptive and inferential statistics, such as percentages, frequencies, means, standard deviations and confidence intervals, were used to present the basic statistics in relation to the demographics, knowledge, health beliefs and illness perception variables. Tests for associations (Chi-square test: to determine significant associations between one categorical variable and another categorical variable) were applied to investigate the associations of health beliefs, illness representations, knowledge, socio-demographic factors and health status with uptake to MBSP. Binary logistic regression modelling, using the “Backward-elimination” method, was performed to identify the significant predictors for BS uptake. The unstandardized coefficients, standard error, the Wald value, *p*-values, Odds Ratios (ORs) and 95% confidence intervals (95% CIs) were calculated for each logistic regression model. The level of accuracy was included in the final outcome of the model. Missing data was minimal and reported in Table 1. Statistical significance was established at *p* < 0.05 for all analyses.

**Table 1:** Sample Characteristics (*n* = 404)

Characteristics	Mean	SD	N	%
Age (year)				
50			20	5.0
51			45	11.1
52			42	10.4
53			48	11.9
54			56	13.9
55			44	10.9
56			29	7.2
57			44	10.9
58			27	6.7
59			37	9.2
60			12	3.0
	54.62	2.79		
Education level				
No schooling			1	0.3
Primary level			67	16.6
Secondary level			306	75.7
Tertiary level			30	7.4
Occupation				
Pensioner			5	1.2
Housewife			311	77.0
Employee			88	21.8
Status				
Single			16	4.0
Married			351	86.9
Separated/Divorced			13	3.2
Widowed			24	5.9
Family income				
Less than €10,737			102	25.3
€10,737 – €16,113			142	35.2
€16,114 – €23,563			20	5.0
€23,564 – €33,966			14	3.5
Greater than €33,966			1	0.3
Prefer not to say			125	30.9
Own a car				
Yes			338	83.7
No			66	16.3
Drive				
Yes			177	43.8
No			227	56.2
Any illness, disability or condition				
Yes			185	45.8
No			219	54.2
Family physician (GP)				
Yes			377	93.3
No			27	6.7
Frequency of GP visit				
Only when I have a problem			358	88.6
Once a month			6	1.5
More than once a year			16	4.0
Once a year			1	0.2
Missing			23	5.7
Lumpy breasts				
Yes			30	7.4
No			374	92.6
Relatives or close friends had cancer				
Yes			330	81.7
No			68	16.8
Prefer not to say			6	1.5

### Piloting the data collection method

A pilot study was conducted with a random sample of 15 women of different age groups to assess and ascertain the practicalities of conducting the tool by telephone. In order to reduce bias, a random selection of participants was computer generated from the computerized database of the MBSP; hence, attendance for first round screening was ascertained from programme records. A similar approach to the larger study for ‘selection’ and ‘recruitment’ can be similarly referred to in the participant pathway (Fig. 1). These women were contacted by a research assistant and those who agreed to participate were introduced to the researcher. A convenient time was arranged with each participant in order for the researcher to conduct the pilot survey by telephone. Verbal informed consent was sought from all 15 participants. The results from the pilot study showed that the tool was practical and feasible to conduct by telephone and that no methodological changes were required. Women participating in the pilot study were not included in the larger study. The time for scale completion had a median of 25 min (range, 15–45 min).

## Results

### Sample characteristics

The sample characteristics (*n* = 404) are presented in Table 1. Women were aged between 50 to 60 years at the time of the programme’s first screening round, with a mean age of 54.6 years ±2.8 years (SD). The majority were married (86.9%, *n* = 351), housewives (77%, *n* = 311), had up to a secondary education level (75.7%, *n* = 306) and more than half (60.3%, *n* = 244) were from below average income families (lower than €16,113). Although the majority owned a family car (83.7%, *n* = 338), only 43.8% (*n* = 177) could drive. An illness, disability or condition was reported by 45.8% of women (*n* = 185) and 2.5% (*n* = 10) had cancer (other than BC). Furthermore, 81.7% (*n* = 330) had relatives or close friends with cancer [6.7% (mother with BC) and 21.3% (close friend with BC)]. The majority (93.3%, *n* = 377) reported having a named family physician (GP); however, 88.6% (*n* = 358) of the total sample visited a GP only when they had a problem. Furthermore, nearly 70% of women in this study reported that they were not encouraged by their GP to attend to breast screening.

### Knowledge of breast screening frequency and breast cancer

The majority of women were knowledgeable of the recommended screening frequency to varying degrees (Table 2): 46.3% (*n* = 187) indicated yearly mammograms; 3.7% (*n* = 15): every 1.5 years; 43.3% (*n* = 175): every 2–3 years; 6.2% (*n* = 25) were unsure). BC identity scores were reported by above 80% of women for the majority of the sub-scale items (7 out of 8 items) (Table 3). However, there was wide variation for knowledge of causes and risk factors of BC among Maltese women (Table 3). Hereditary predisposition to the disease was the most commonly reported risk factor, followed by smoking, altered immunity and pollution. Misconceptions concerning risk factors of BC were found [e.g. a germ or virus (38.6% ‘agree’, 30.7% ‘disagree’; accident or injury (47.5% ‘agree’; 39.1% ‘disagree’)].

**Table 2:** Women’s Knowledge of breast screening frequency (*n* = 404)

	n	%
Knowledge about recommended breast screening frequency		
Every year	187	46.3
Every year and a half	15	3.7
Every 2–3 years	175	43.3
Every 4–5 years	2	0.5
Unsure	25	6.2

**Table 3:** Women’s Knowledge on breast cancer identity and causes (*n* = 404)

Breast cancer identity scores, n (%)			
	Disagree/Strongly Disagree	Undecided	Agree/Strongly Agree
The presence of a lump or thickening in the breast	5 (1.2)	26 (6.4)	373 (92.3)
Nipple discharge	3 (0.7)	54 (13.4)	347 (85.9)
Sudden nipple retraction	2 (0.5)	64 (15.8)	338 (83.7)
Change in shape or appearance of the nipple	2 (0.5)	29 (7.2)	373 (92.3)
Breast swelling, dimpling, redness or soreness of the skin	3 (0.7)	66 (16.3)	335 (82.9)
Skin changes of the breast	3 (0.7)	67 (16.6)	334 (82.7)
A sudden change in breast size	5 (1.2)	52 (12.9)	347 (85.9)
Aching breasts	40 (9.9)	114 (28.2)	250 (61.9)
Causes of breast cancer scores, n (%)			
	Disagree/Strongly Disagree	Undecided	Agree/Strongly Agree
Stress or worry	152 (37.6)	95 (23.5)	157 (38.9)
Your mental attitude	262 (64.9)	94 (23.3)	48 (11.8)
Family problems or worries	171 (42.3)	82 (20.3)	151 (37.4)
Overwork	281 (69.6)	59 (14.6)	64 (15.8)
Your emotional state	257 (63.6)	76 (18.8)	71 (17.6)
Your personality	262 (64.9)	94 (23.3)	48 (11.8)
Hereditary - it runs in the family	5 (1.2)	10 (2.5)	389 (96.3)
Diet or eating habits	121 (30.0)	61 (15.1)	222 (55.0)
Poor medical care in the past	98 (24.3)	90 (22.3)	216 (53.4)
Your own behaviour	174 (43.1)	172 (42.6)	58 (14.3)
Ageing	142 (35.1)	63 (15.6)	199 (49.3)
Smoking	47 (11.6)	39 (9.7)	318 (78.7)
Alcohol	80 (19.8)	60 (14.9)	264 (65.3)
A germ or virus	124 (30.7)	124 (30.7)	156 (38.6)
Pollution in the environment	65 (16.1)	49 (12.1)	290 (71.8)
Altered immunity	43 (10.6)	69 (17.1)	292 (72.3)
Chance or bad luck	205 (50.7)	37 (9.2)	162 (40.1)
Accident or injury	158 (39.1)	54 (13.4)	192 (47.5)

### Health beliefs and illness perceptions

Women’s health beliefs and illness perceptions are presented in Table 4. Subscale scores were retrieved as the mean of items (i.e. those items with which respondents are most in agreement, though a disagreement answer for barrier items represents a more positive result). In general, higher percentage scores indicate higher agreement among participants for perceived benefits of mammography (79.7%), self-efficacy (77.7%) and cues to action (76.6%), while lower scores indicate lower agreement among women for perceived barriers (45.1%). There was also higher agreement with emotional representations (82.0%), personal control items (78.7%), BC identity (76.5%) and cyclical cancer timeline perceived (72.0%), while lower agreement for BC causes (62.4%) and cancer timeline (acute/chronic) (61.0%).

**Table 4:** Instrument scoring: the percentage and mean scores for Health Beliefs and Illness Perceptions

Health Beliefs				
^a^Subscale	Minimum	Maximum	Mean Score	Percentage Score
Perceived Susceptibility	3	15	9.6	64.0%
Perceived Benefits	6	30	23.9	79.7%
Perceived Barriers	13	65	29.3	45.1%
Cues to action	7	35	26.8	76.6%
Self-Efficacy	7	35	27.2	77.7%
Illness Perceptions				
^a^Subscale	Minimum	Maximum	Mean Score	Percentage Score
Breast Cancer Identity	8	40	30.6	76.5%
Causes of Breast Cancer	18	90	56.2	62.4%
Cancer Timeline: Acute/Chronic	2	10	6.1	61.0%
Cancer Timeline: Cyclical	1	5	3.6	72.0%
Consequences	8	40	28.3	70.8%
Personal Control	3	15	11.8	78.7%
Treatment Control	3	15	9.9	66.0%
Illness Coherence	2	10	7	70.0%
Emotional Representations	5	15	12.3	82.0%

^a^All subscale items were grouped according to their respective subscale. Each subscale item had 5 response options ranging from 1 = ‘strongly disagree’ to 5 = ‘strongly agree’

When comparing health beliefs and illness perceptions among attendees and non-attendees (Tables 5– 6), the majority agree that the possibility of developing BC in their lifetime is high (M = 4.0, SD = 0.3) and believe in early detection through screening (M = 4.2, SD = 0.5). Each item in the ‘perceived barrier’ subscale was scored by respondents with the highest level of uncertainty, such that 6 out of 13 items had a mean score of 2.5–3.5 (Table 5).

**Table 5:** Comparison of Health Beliefs between attendees and non-attendees

When you received your invite to the Breast Screening programme, did you attend?	Yes		No		Total		Chi-Square test^a^	
Health Beliefs	Mean	SD	Mean	SD	Mean	SD	χ2	*p*-value
There is no possibility of getting breast cancer *(r)*	1.9	0.7	2.0	0.7	2.0	0.7	4.3	0.367
Your chances of getting breast cancer are high	3.7	0.7	3.6	0.8	3.6	0.8	7.1	0.130
There may be the possibility of developing breast cancer in your lifetime	4.0	0.3	4.0	0.4	4.0	0.3	1.7	0.645
When you get a mammogram, you feel good about yourself	4.0	0.4	3.9	0.5	4.0	0.5	16.7	0.001*
When you get a mammogram, you do not worry as much about breast cancer	3.8	0.8	3.6	0.8	3.7	0.8	2.8	0.423
Having a mammogram will help you find lumps early in your breasts	4.2	0.4	4.1	0.5	4.2	0.5	7.8	0.051
If you find a lump through a mammogram, the treatment for breast cancer may not be as bad	4.0	0.4	4.0	0.3	4.0	0.4	3.3	0.349
Having a mammogram will decrease your chances of dying from breast cancer	4.0	0.4	4.0	0.3	4.0	0.3	6.2	0.103
Having a mammogram will help you find a lump before it can be felt by yourself or a health professional	4.0	0.5	4.0	0.4	4.0	0.5	0.6	0.899
Having a routine mammogram would make you anxious about breast cancer	2.7	1.0	2.9	1.0	2.8	1.0	7.1	0.070
Having a routine mammogram would make you worry	2.7	1.0	2.9	1.0	2.8	1.0	3.9	0.416
You fear having a mammogram because you might find out that something is wrong	2.9	1.0	3.2	1.0	3.0	1.1	12.0	0.017*
You fear having a mammogram because you do not know the procedure or what to expect	2.2	0.6	2.5	0.9	2.3	0.8	31.9	0.000*
You fear having a mammogram because you know someone (family or friend) with breast cancer	2.6	1.1	2.9	1.1	2.7	1.1	7.1	0.132
It is embarrassing for you to have a mammogram	2.4	0.8	2.7	1.0	2.5	0.9	13.6	0.009*
Undergoing mammography will be painful or uncomfortable	3.4	1.0	3.3	0.9	3.3	1.0	39.0	0.000*
Having a mammogram is time consuming	1.2	0.4	1.3	0.6	1.3	0.5	7.2	0.067
You are discontent with Breast Screening personnel as they have been rude to you	1.2	0.5	n/a	n/a	1.2	0.5	n/a	n/a
You have fear or distrust in the medical team	1.7	0.7	2.2	0.9	1.9	0.8	38.3	0.000*
Having a mammogram would expose you to unnecessary radiation	2.2	0.6	2.5	0.8	2.3	0.7	16.6	0.001*
You have too many other problems in your life than to get a mammogram done	1.6	0.6	2.0	0.8	1.7	0.7	38.8	0.000*
You are not old enough to have a mammogram periodically	1.7	0.5	1.9	0.4	1.8	0.5	22.6	0.000*
If your GP advises you to attend for a mammogram, you will attend	4.3	0.6	4.0	0.7	4.2	0.7	13.6	0.004*
If your relatives or friends advise you to attend for a mammogram, you will attend	3.4	1.0	3.4	1.0	3.4	1.0	2.0	0.576
If someone close to you has been diagnosed with breast cancer, you will attend for a mammogram	4.2	1.0	3.9	1.0	4.1	1.0	13.8	0.008*
Hearing about breast cancer and breast screening in the media or news makes you think about getting a mammogram	3.8	0.7	3.5	0.9	3.6	0.8	15.7	0.000*
Reminder letters would help you to get a mammogram	4.0	0.4	3.8	0.7	3.9	0.5	15.4	0.001*
Reminder phone calls or text messages would help you to get a mammogram	4.0	0.4	3.8	0.7	3.9	0.5	15.4	0.001*
Routine educational talks regarding breast cancer awareness would help you to get a mammogram	3.8	0.7	3.5	0.9	3.6	0.8	16.9	0.001*
You feel confident that if you had a mammogram done, any abnormalities in your breasts will be detected	3.7	0.6	3.6	0.7	3.7	0.6	2.2	0.697
You can arrange other things in your life to get a mammogram	4.2	0.6	4.0	0.7	4.1	0.7	13.1	0.011*
In case you need a mammogram, you will find a place to get it done	4.2	0.5	4.1	0.5	4.2	0.5	10.9	0.028*
You can make an appointment for a mammogram	4.2	0.5	4.1	0.6	4.2	0.5	12.1	0.016*
You can arrange transportation to get a mammogram	4.2	0.5	4.1	0.6	4.2	0.6	13.1	0.011*
You can talk to people at the breast screening centre about your concerns	4.1	0.7	n/a	n/a	4.1	0.7	n/a	n/a
You can find a way to pay for a mammogram if you need to	4.2	0.5	4.1	0.5	4.2	0.5	10.3	0.036*

*Significant at α = 0.05

*(r)* = *reverse scored*

^a^Chi-square test was applied for all health beliefs; hence the categorical answers were used to apply this test for association. For each question, respondents were asked to select a number between 1 and 5, where 1 = strongly disagree and 5 = strongly agree.

**Table 6:** Comparison of Illness Perceptions between attendees and non-attendees

When you received your invite to the Breast Screening programme, did you attend?	Yes		No	Total			Chi-Square test^a^	
Illness Perception	Mean	SD	Mean	SD	Mean	SD	χ2	*p*-value
The presence of a lump of thickening in the breast	3.9	0.4	3.9	0.3	3.9	0.3	5.5	0.141
Nipple discharge	3.9	0.4	3.8	0.4	3.9	0.4	3.8	0.286
Sudden nipple retraction	3.9	0.4	3.8	0.4	3.8	0.4	5.8	0.121
Change in shape or appearance of the nipple	3.9	0.3	3.9	0.3	3.9	0.3	1.7	0.630
Breast swelling, dimpling, redness or soreness of the skin	3.8	0.4	3.8	0.4	3.8	0.4	2.6	0.463
Skin changes of the breast	3.8	0.4	3.8	0.4	3.8	0.4	2.1	0.555
A sudden change in breast size	3.8	0.4	3.9	0.4	3.9	0.4	0.4	0.950
Aching breasts	3.5	0.7	3.5	0.7	3.5	0.7	2.9	0.578
Stress or worry	3.0	0.9	3.0	0.8	3.0	0.9	3.9	0.140
Your mental attitude (e.g. thinking about life negatively)	2.5	0.7	2.5	0.7	2.5	0.7	6.0	0.111
Family problems or worries	3.0	0.9	2.9	0.9	3.0	0.9	3.5	0.178
Overwork	2.5	0.8	2.4	0.7	2.5	0.8	4.1	0.249
Your emotional state (e.g. feeling down, lonely, anxious, empty)	2.5	0.8	2.6	0.8	2.5	0.8	19.0	0.000*
Your personality	2.4	0.7	2.5	0.7	2.5	0.7	6.6	0.087
Hereditary - it runs in the family	4.6	0.6	4.4	0.6	4.5	0.6	13.4	0.004*
Diet or eating habits	3.3	0.9	3.2	0.9	3.3	0.9	5.6	0.131
Poor medical care in the past	3.3	0.8	3.3	0.8	3.3	0.8	2.4	0.489
Your own behaviour	2.7	0.7	2.8	0.7	2.7	0.7	10.0	0.018*
Ageing	3.1	0.9	3.1	0.9	3.1	0.9	4.9	0.087
Smoking	3.7	0.7	3.6	0.7	3.7	0.7	3.0	0.399
Alcohol	3.5	0.8	3.4	0.8	3.5	0.8	0.1	0.948
A germ or virus	3.0	0.8	3.1	0.8	3.1	0.8	3.7	0.160
Pollution in the environment	3.7	0.8	3.5	0.8	3.6	0.8	6.1	0.108
Altered immunity	3.6	0.7	3.6	0.7	3.6	0.7	1.5	0.683
Chance or bad luck	3.0	1.0	2.8	0.9	2.9	1.0	5.8	0.214
Accident or injury	3.1	0.9	3.1	0.9	3.1	0.9	1.7	0.782
Breast cancer will last a short time	2.8	0.7	2.9	0.7	2.8	0.7	4.2	0.241
Breast cancer is likely to be permanent rather than temporary	3.3	0.8	3.2	0.8	3.3	0.8	1.5	0.481
A patient with breast cancer goes through cycles in which her illness gets better and worse	3.7	0.7	3.4	0.7	3.6	0.7	11.1	0.026*
Breast cancer has major consequences on a patient's life	4.3	0.6	4.2	0.5	4.3	0.6	14.2	0.003*
Breast cancer will not have much effect on your life	1.5	0.7	1.6	0.7	1.5	0.7	11.8	0.019*
Breast cancer would strongly affect the way others see you	3.3	1.0	3.3	0.9	3.3	0.9	14.9	0.005*
Breast cancer has serious economic and financial consequences	3.9	0.6	3.7	0.7	3.8	0.6	13.3	0.004*
Breast cancer would strongly affect the way you see yourself as a person	4.1	0.5	4.0	0.6	4.1	0.6	0.7	0.875
Breast cancer would threaten a relationship with your husband or partner	3.1	0.9	3.0	0.9	3.1	0.9	2.2	0.699
If you had breast cancer, your whole life would change	4.3	0.7	4.1	0.6	4.2	0.6	18.0	0.000*
If you developed breast cancer, the chances of living a long life would decrease	4.0	0.5	4.0	0.3	4.0	0.4	9.4	0.024*
There is a lot which you can do to control the symptoms if Breast Cancer occurs	3.9	0.4	3.9	0.4	3.9	0.4	2.6	0.629
The course of Breast Cancer will depend on your actions	4.0	0.4	3.9	0.3	3.9	0.4	5.9	0.118
Your actions will have an effect on the outcome of Breast Cancer	4.0	0.3	4.0	0.2	4.0	0.3	5.9	0.118
There is no treatment that will help to improve Breast Cancer	2.0	0.6	2.0	0.5	2.0	0.5	5.8	0.211
The treatment provided will be effective in controlling or curing Breast Cancer	4.0	0.3	3.9	0.3	4.0	0.3	1.8	0.615
The negative effects of Breast Cancer can be prevented or avoided by the treatment given	4.0	0.3	3.9	0.3	3.9	0.3	5.5	0.241
You have a clear picture and understanding of Breast Cancer	3.8	0.6	3.8	0.6	3.8	0.6	0.7	0.873
Breast Cancer is a mystery to you	3.2	1.0	3.2	1.0	3.2	1.0	3.7	0.455
You get anxious when you think about Breast Cancer	3.6	1.1	3.7	1.1	3.6	1.1	2.6	0.464
Breast Cancer makes you feel afraid	4.3	0.7	4.3	0.6	4.3	0.7	1.7	0.645
You get worried when you think about Breast Cancer	4.4	0.7	4.4	0.6	4.4	0.7	1.4	0.502

*Significant at α=0.05

^a^Chi-square test was applied for all illness perceptions; hence the categorical answers were used to apply this test for association. For each question, respondents were asked to select a number between 1-5, where 1 = strongly disagree and 5 = strongly agree.

This study found that a large number of participants had higher emotional representations when they think about BC, such that they get anxious (M = 3.6, SD = 1.1), feel afraid (M = 4.3, SD = 0.7) and worried (M = 4.4, SD = 0.7), they believe that BC has major consequences on a patient’s life (M = 4.3, SD = 0.6), and more specifically, their whole life would change (M = 4.2, SD = 0.6). The course of the BC pathway is believed to be dependent on their actions (M = 3.9, SD = 0.4).

### Reasons for non-attendance to first breast screening invitation

When non-attendees were asked to further identify reasons for non-attendance to first round screening at the MBSP (i.e. respondents were allowed to mention more than one reason), the main reported reason was fear (41.0%, *n* = 66), of which sub-categories included ‘fear of result’ (20.5%; *n* = 33), ‘fear of pain’ (10.6%; *n* = 17), ‘fear of an unknown procedure’ (depicting knowledge gap) (6.2%; *n* = 10), ‘fear of radiation’ (3.7%, *n* = 6) and ‘embarrassment’ (8.1%; *n* = 13). Some women had also opted for the service elsewhere (38.5%, *n* = 62) or had never received an invitation (13.7%; *n* = 22). Practical reasons were mentioned by 8.7% (*n* = 14) of non-attendees, which included ‘busy at work’ or ‘home’, ‘transport issues’, ‘on vacation’ and ‘being ill’.

### Associations between health beliefs and uptake to first screening invitation

The variables related to HBM constructs were compared with attendance and non-attendance to the first round screening at the MBSP (Table 5). In general, the majority of the HBM constructs showed statistical significance as follows:

### Perceived benefits

Women who feel good about themselves when getting a mammogram (χ2 = 16.7, *p* = 0.001) were more likely to attend their first screening invitation. On the other hand, non-attendees believe less than attendees that BS will help to detect a lump early before it can be felt (χ2 = 7.8, *p* = 0.051).

### Perceived barriers

Although there was no significant association between anxiety and initial screening uptake, fear was found to be statistically significant across all subscale items (*p* < 0.05). Non-attendees expressed fear of a cancer diagnosis (χ2 = 12.0, *p* = 0.017), fear of the unknown procedure (χ2 = 31.9, *p* = 0.000), fear of radiation (χ2 = 16.6, *p* = 0.001), consider mammography to be embarrassing (χ2 = 13.6, *p* = 0.009) and other problems in life to be greater than getting a mammogram (χ2 = 38.8, *p* = 0.000), and were more undecided on whether the mammography procedure is painful (χ2 = 39.0, *p =* 0.000). On the other hand, attendees are more in disagreement with the statement: ‘they are not old enough to have a mammogram periodically’ (χ2 = 22.6, *p* = 0.000) and have less fear or distrust in the medical team (χ2 = 38.3, *p* = 0.000).

### Cues to action

Women attend more if advised by their GP (χ2 = 13.6, *p =* 0.004) and if someone close to them had BC (χ2 = 13.8, *p* = 0.008), but do not attend more if advised by their relatives or friends (χ2 = 2.0, *p* = 0.576). Attendees are more in agreement that hearing about BC and BS in the media or news makes them think about getting a mammogram (χ2 = 15.7, *p* = 0.000), and similarly reminder letters (χ2 = 15.4), phone calls or text messages (χ2 = 15.4), and educational talks (χ2 = 16.9) help them to get a mammogram done (*p* = 0.001 respectively).

### Self-efficacy

Attendees also tend to agree more that they can arrange other things in life to get a mammogram (χ2 = 13.1, *p* = 0.011), such as finding a place to get it done (χ2 = 10.9, *p* = 0.028), arranging an appointment (χ2 = 12.1, *p* = 0.016) and transportation (χ2 = 13.1, *p* = 0.011), and also paying for it if they need to (χ2 = 10.3, *p* = 0.036).

### Associations between illness perceptions and uptake to first screening invitation

Illness perception constructs were compared with attendance and non-attendance to the first screening invitation to the MBSP (Table 6). In general, Chi-square tests showed no statistical significance for BC identity items, acute/chronic cancer timeline, personal and treatment control, illness coherence and emotional representation items with first screening uptake.

### Causes of breast cancer

In general, no significant association was found for most causal variables. However, attendees were more in agreement that BC could be hereditary (χ2 = 13.4, *p* = 0.004) and considered one’s own behaviour to cause BC (χ2 = 10.0, *p* = 0.018), while non-attendees were more undecided whether one’s emotional state or personality cause BC (χ2 = 19.0, *p* = 0.000).

### Cancer timeline (cyclical)

Attendees agree more than non-attendees that a patient with BC gets better and worse (χ2 = 11.1, *p* = 0.026).

### Consequences

Attendees consider more that BC has major consequences on a patient’s life (χ2 = 14.2, *p* = 0.003), has serious economic and financial consequences (χ2 = 13.3, *p* = 0.004) and is life-changing (χ2 = 18.0, *p* = 0.000). On the other hand, non-attendees are more undecided whether BC would strongly affect the way others see them (χ2 = 14.9, *p* = 0.005) and consider the chances of living a long life to decrease (χ2 = 9.4, *p* = 0.024).

### Associations between sociodemographic and health status, knowledge of breast screening frequency and uptake to first screening invitation

There were no significant associations for demographic factors or health status variables with first screening uptake, except for family income (χ2 = 9.7, *p* = 0.047). Non-attendees were the most unsure of the recommended screening frequency (χ2 = 13.9, *p =* 0.003).

### Predictors of uptake to first screening invitation

Different groups of variables and constructs were incorporated into seven logistic regression models and the ‘backward-elimination’ method was applied to every model to identify the significant predictors of BS uptake (Table 7).

**Table 7:** Comparison of Illness Perceptions between attendees and non-attendees

	B	SE	Wald	*P*-value	OR	95% CI	Model Accuracy YES	Model Accuracy NO
Model 1: Demographics							100%	0%
Drive	-0.361	0.207	3.047	0.081	0.697	0.465, 1.045		
Constant	0.979	0.342	8.172	0.004	2.661			
Model 2: Health Status							100%	0%
Breast condition	0.174	0.265	0.430	0.512	1.190	0.708, 1.998		
Constant	0.081	0.492	0.027	0.869	1.085			
Model 3: Health Beliefs							88.5%	38.8%
Distrust in medical team	−0.573	0.153	14.051	0.000	0.564	0.418, 0.761		
Fear of unknown procedure	−0.409	0.153	7.120	0.008	0.664	0.492, 0.897		
Other life problems	−0.693	0.195	12.630	0.000	0.500	0.341, 0.733		
Relatives or friends’ advice	−0.363	0.130	7.745	0.005	0.696	0.539, 0.898		
Reminder letters	0.660	0.238	7.678	0.006	1.934	1.213, 3.083		
Constant	2.336	1.091	4.585	0.032	10.335			
Model 4: Illness Perceptions							83.5%	37.3%
Hereditary	0.456	0.185	6.072	0.014	1.578	1.098, 2.268		
Pollution	0.290	0.134	4.682	0.030	1.336	1.028, 1.738		
Illness gets better and worse	0.312	0.153	4.154	0.042	1.366	1.012, 1.844		
Major consequences in life	0.420	0.195	4.640	0.031	1.522	1.039, 2.231		
Whole life would change	0.509	0.201	6.442	0.011	1.664	1.123, 2.466		
Living long decreases	−0.685	0.298	5.290	0.021	0.504	0.281, 0.904		
Fear of breast cancer	−0.363	0.176	4.264	0.039	0.695	0.492, 0.983		
Constant	−3.375	1.494	5.106	0.024	0.034			
Model 5: Health Beliefs and Illness Perceptions							84.8%	53.8%
Distrust in medical team	−0.676	0.162	17.468	0.000	0.509	0.371, 0.699		
Fear of unknown procedure	−0.612	0.166	13.629	0.000	0.542	0.392, 0.751		
Other life problems	−0.669	0.206	10.544	0.001	0.512	0.342, 0.767		
Relatives or friends’ advice	−0.476	0.140	11.610	0.001	0.621	0.473, 0.817		
Reminder letters	0.687	0.251	7.470	0.006	1.987	1.214, 3.251		
Pollution	0.479	0.151	10.060	0.002	1.615	1.201, 2.172		
Illness gets better and worse	0.396	0.167	5.656	0.017	1.486	1.072, 2.061		
Whole life would change	0.855	0.221	14.924	0.000	2.351	1.524, 3.626		
Living long decreases	−0.890	0.336	7.016	0.008	0.411	0.212, 0.793		
Constant	0.113	1.742	0.004	0.948	1.120			
Model 6: Health Beliefs and Illness Perceptions							85.2%	65.0%
Fear of unknown procedure	−0.742	0.194	14.633	0.000	0.476	0.325, 0.696		
Embarrassing	−0.320	0.149	4.600	0.032	0.726	0.542, 0.973		
Distrust in medical team	−0.808	0.176	21.149	0.000	0.446	0.316, 0.629		
Other life problems	−0.735	0.234	9.843	0.002	0.479	0.303, 0.759		
Relatives or friends’ advice	−0.529	0.153	11.965	0.001	0.589	0.437, 0.795		
Reminder letters	0.795	0.290	7.536	0.006	2.215	1.255, 3.907		
Arrange appointment	1.133	0.506	5.020	0.025	3.106	1.153, 8.372		
Pay for mammography	−1.669	0.580	8.286	0.004	0.188	0.06, 0.587		
Stress or worry	−0.940	0.419	5.044	0.025	0.39	0.172, 0.887		
Family problems	0.839	0.405	4.292	0.038	2.314	1.046, 5.118		
Overwork	0.539	0.216	6.262	0.012	1.715	1.124, 2.616		
Personality	−0.548	0.240	5.235	0.022	0.578	0.361, 0.924		
Hereditary	0.533	0.231	5.342	0.021	1.704	1.084, 2.677		
Pollution	0.500	0.170	8.698	0.003	1.649	1.183, 2.299		
Change or bad luck	0.432	0.140	9.568	0.002	1.54	1.171, 2.024		
Illness gets better and worse	0.398	0.185	4.629	0.031	1.489	1.036, 2.141		
Economic consequences	0.647	0.223	8.438	0.004	1.91	1.234, 2.955		
Whole life would change	0.755	0.245	9.493	0.002	2.128	1.316, 3.441		
Living long decreases	−1.177	0.373	9.956	0.002	0.308	0.148, 0.64		
Depends on your actions	0.856	0.409	4.381	0.036	2.354	1.056, 5.246		
Your actions effects outcome	−1.094	0.552	3.933	0.047	0.335	0.114, 0.987		
Constant	0.384	3.083	0.016	0.901	1.468			
Model 7: The 14 constructs							84.4%	42.2%
Perceived barriers	−0.121	0.022	31.731	0.000	0.886	0.849, 0.924		
Cancer timeline cyclical	0.432	0.154	7.893	0.005	1.54	1.139, 2.081		
Illness coherence	0.249	0.100	6.179	0.013	1.283	1.054, 1.561		
Constant	0.623	0.895	0.484	0.487	1.864			

*B* unstandardized coefficients; *SE* standard error; *OR* odds ratio; *CI* confidence interval

### Model 1 (Demographics) and Model 2 (Health status)

All items related to demographic variables were incorporated in a logistic regression model (Model 1) and health status items were incorporated into Model 2. Both demographics and health status variables were found to be non-important predictors of BS uptake, such that for both models, non-attendance was not predicted and none of the variables were found to be significantly different.

### Model 3 (Health Belief items)

All items related to HBM were incorporated in a logistic regression model (Model 3). Five variables were found to be good predictors of BS uptake: ‘distrust in medical team’, ‘fear of unknown procedure’, ‘other life problems’, ‘relatives and friends̕ advice’ and ‘reminder letters’ (Table 7). For this model, attendance was predicted with an accuracy of 88.5% and non-attendance was predicted with 38.8%.

### Model 4 (Illness Perception items)

All IPQ-R variables were incorporated into one logistic regression model (Model 4). Seven variables were found to be good predictors: ‘hereditary’, ‘pollution’, ‘a patient with BC goes through cycles in which her illness gets better and worse’, ‘BC has major consequences on a patient’s life’, ‘if you had BC, your whole life would change’, ‘if you developed BC, the chances of living a long life would decrease’ and ‘BC makes you feel afraid’ (Table 7). The accuracy for this model was found to be 83.5% for attendance and 37.3% for non-attendance.

### Model 5 (Significant predictors from Models 3 and 4)

The above significant predictors from both models 3 and 4 were incorporated into a new single model (Model 5) and backward-elimination was applied on these 12 variables (five Health Beliefs and seven Illness Perception variables). The final model retained nine significant predictors, without excluding any of the Health Belief variables, hence showing that Health Beliefs are more significant predictors than Illness Perceptions. The model accuracy, when combining both scores, improved to 53.8% for non-attendance and 84.8% for attendance.

### Model 6 (All individual Health Belief and Illness Perception items)

When all items related to Health Beliefs and Illness Perceptions were incorporated into one model (Model 6), 21 variables were found to be significantly different. The accuracy of the model improved again to 85.2% for attendees and 65% accuracy for non-attendees.

### Model 7 (All 14 constructs)

When the 14 constructs (not individual items) related to Health Beliefs and Illness Perceptions were used to construct a logistic regression model (Model 7), ‘perceived barriers’, ‘cancer timeline (cyclical)’ and ‘illness coherence’ were found to be the significant predictors, of which the ‘perceived barriers’ construct was the strongest predictor. However, the accuracy for predicting the non-attendees was found to be 42.2%, which is inferior when compared to Model 5. Moreover, when removing the ‘perceived barriers’ variable from the latter model, the accuracy to predict non-attendance decreased sharply from 42.2% to 14.9%.

Our findings reveal that ‘perceived barriers’ is the most important construct to describe the variance between attendees and non-attendees. This result is further echoed in Model 5, where three predictors (from all the other predictors) are all related to perceived barriers. The above logistic regression analyses show that, although Health Beliefs are the most important predictors of BS uptake, the inclusion of Illness Perception items into one logistic regression model is important to improve the accuracy of the model (Model 5 vs Model 3).

## Discussion

For the first time, this study aimed to explore factors related to Maltese women’s BS behaviours, as well as their knowledge, health beliefs and illness perceptions related to BC and BS, providing answers as to why more than 40% of eligible women did not attend their first MBSP invitation.

### Knowledge

Study findings confirm the wide variation in knowledge level of Maltese women about causes of BC and its related risk factors, though good awareness of BC signs and symptoms were reported, such as nipple discharge and sudden nipple retraction. Women’s limited knowledge about BC and BS practices has been identified in a consistent body of literature [22, 32, 44, 45]. For instance, Grunfeld et al. [46] showed that only 38% of people were aware that nipple retraction was a sign of BC, and awareness of risk factors was even lower. Notably, local misconceptions (e.g., one’s own behaviour, personality, emotional state, germ or virus, accident or injury could cause BC) also corroborate findings in older studies (e.g., hitting or bumping the breast), which is consistent with women’s beliefs in other societies with different cultures such as the Philippines, Korea, Saudi Arabia and Australia [45–47].

Since relevant knowledge has been emphasized as a screening compliance predictor [48, 49] or a screening barrier [50], we hypothesized that there would be a significant association between knowledge and BS uptake in Malta. Our findings support this hypothesis since Maltese women who have a lack of awareness regarding screening recommendations, guidelines and BC related risk factors are more likely not to attend and this may prove difficult for women to perceive their risk [22]. Communicating risk information to the general public makes knowledge an essential element of health promotion, disease prevention and screening interventions [51]. Despite the vast array of worldwide initiatives, an overlap exists between knowledge, health beliefs and illness perceptions; the knowledge construct operationalized in BS studies does not often include identifications of specific beliefs [48]. Hence, in order for a woman to attend for her BS appointment, she must perceive the actual threat of BC, believe that cancer can be avoided by BS, and that she is capable of accessing the unit, which may include remembering her appointment, driving to or be driven to the unit, and not be afraid of the test [52].

### Reasons to non-attendance

Fears, negative expectation of the screening experience and embarrassment were among the main barriers to BS in this study, similarly reported to act as barriers to attendance and re-attendance worldwide [15, 16, 45, 49, 53–59]. Minor practical barriers to non-attendance reported in our study (such as lack of time, transportation issues) are also reiterated in previous studies [16, 56, 58, 59], justifying local transportation accessibility improvements and reduction of logistical barriers [32].

### Health beliefs and illness perceptions

Significant associations were mainly found for health beliefs about BS and BC i.e. the perceptions of the behaviour (barriers, self-efficacy, cues to action), while weaker associations were found for the perceptions of the illness i.e. significant associations for certain illness perception items (causes, cyclical cancer timeline, consequences) with uptake. Non-attendance to BS was related to more perceived barriers, less perceived benefits, lower self-efficacy and cues to action, and to the representations of the causes, consequences and timeline of BC. In contrast to HBM, perceived susceptibility was not significantly associated with first screening attendance in this study; a finding which corroborates results in previous studies [60–62] and contrasts others [18, 23, 62, 63]. One explanation for this finding may be due to women’s lack of knowledge about BC and BS [60], such that improving women’s risk assessment of developing BC may increase uptake rates. Our findings are in agreement with previous studies where positive association with perceived self-efficacy and having BS was found [62, 63]. This implies that attendees feel confident that they can arrange other things in their life to get a mammogram. However, self-efficacy was not the most important predictor for the decision to undergo screening in Malta. This result complies with a study in Cyprus [64] and contrasts the findings by Orji et al. [29].

It has also been reported that if a woman perceives mammography benefits to be higher than perceived barriers, she is more likely to adhere to BS [23, 58, 65]. However, the benefits subscale was not the most significant component associated with BS in Malta unlike in other studies [53, 66]. It was the strong negative association between perceived barriers and screening uptake which was mainly identified in this study, similar to the findings of other studies involving American asymptomatic women [67], Israeli women [68] and other populations [61–63, 69]. It was predominantly fear that was found to be statistically significant across all subscale items. This is evidenced by women who do not attend for mammography in other countries because they perceive greater fear of BC [70–72]. A cancer diagnosis seems to be associated with a negative physical, psychological and social impact on Maltese women’s ability to cope with the outcomes of the disease, which can have a profound effect on their way of life: an economical and financial impact, altered perception of others and oneself, altered relationship with their husband/partner, and that diagnosis may lead to mortality. This is noticeable in other findings [14, 71–73]. It is also likely that the fear of knowing someone with cancer is related to the cultural impact it would have on a woman’s life or social local networks [72]. This consistent fear across populations stems from the belief that there is little an individual can do to alter fate (fatalism) or prevent cancer [73]. Therefore, non-attendees may be more pessimistic of early BC detection and the effectiveness of subsequent treatment, and may perceive BC as being uncontrollable, chronic and highly symptomatic with avoidance and denial coping strategies [74].

Helping to manage barriers associated with cancer and screening could be one of the main tasks addressed by interventions to increase uptake, for example through the use of patient navigators alongside access to care [75] and the identified recommendations from a physician, health care providers, family member and personal communication with other women which have been proven to be of greater importance than external cues [26, 27, 59, 76]. However, our findings are evidence that many women are not encouraged by their GP to attend to BS and would attend more if advised. This is in agreement with a previous study where screening tests are advised at suboptimal rates [59]. Similarly, in a cross-sectional study among Arab women in Qatar, only one quarter of the women interviewed said their doctors had discussed BC with them [77]. It is important to provide a local context for the lack of GP recommendation and to take into account unique aspects of the Maltese health care service. Although the state health system and private GPs provide primary health care in Malta, patients are not affiliated with a regular primary care general practitioner or group practice [78]. Besides, there exists an extent of private purchase of screening outside public health services [78]. However, little is known in Malta regarding the true supporting network of women’s care pathway to date [79]. These issues, coupled with negative women’s representations of BC and perceived barriers to BS may have resulted in non-attendance to first screening invitation at the MBSP.

### Sociodemographic factors and health status

Our findings also demonstrate that women with a lower family income tend to attend less to screening. There is consistent evidence that lower household income demonstrates lower utilization of BS in various countries [16, 54, 80], which also seems to be associated with late stage BC presentation in London [81]. However, in regression analyses, our results revealed that the demographic and health status variables were poor and insignificant predictors of screening uptake and hence, do not provide strength to predict non-attendees. Similarly, sociodemographic factors do not appear to constitute strong predictions of non-attendance in various studies [57], which is why other determinants such as health beliefs and illness perceptions need to be explored within populations because of their importance in stimulating positive health behaviours [53].

### Predictors to first breast screening uptake

Previous studies have demonstrated that beliefs about BC and BS are important predictors of uptake [17, 19, 53, 65, 73]. In our regression analysis, health belief constructs emerged as the strongest and most significant predictors of attendance and non-attendance, and that perceived barriers were the strongest predictor to describe the variance between attendees and non-attendees (*p* < 0.05). This fits well with previous literature, where interventions tailored after the Health Belief Model (HBM) were more effective in increasing BS uptake than those that were not (6 studies OR = 2.51, OR = 1.27, *p* < 0.001) [76]. Limited evidence for the effectiveness of interventions based on other models was found [82].

We found only one Greek study which similarly incorporated both HBM and CSM to explore health beliefs and illness perceptions [17], though this theoretical framework was related to lifetime mammography use as opposed to our study regarding BS uptake in an organised programme. However, their results similarly showed that illness perception dimensions did not prove to be significant predictors of mammography lifetime use. There may be a number of alternative explanations for the non-significant associations and the less significant predictions exhibited by illness representation dimensions and screening uptake in our study. Hagger and Orbell [74] hypothesized that coping may just mediate the effect of illness cognitions on the outcomes of an illness (e.g., psychological well-being, social, and role functioning). This may be due to women’s focus on illness representations (‘mental representation’) as such, rather than on coping strategies (such as obtaining a screening mammogram or visiting a doctor) which, in turn, may possess a different set of diverse and multiple characteristics which IPQ-R does not tackle (e.g., specific beliefs about mammography risks). Therefore, it seems that it is the HBM constructs related to response efficacy (expecting that a particular health action will result in an outcome, such as undertaking mammography screening), self-efficacy, and utility beliefs (believing that taking a certain action would be worthwhile to reduce BC susceptibility or severity, if the disease did occur, while perceived benefits would outweigh perceived barriers to undertaking health actions) that are significant predictors to BS uptake, rather than the IPQ-R dimensions. However, the CSM is the only model which seriously considers the role of emotions in response to illness [83], although even here ‘emotions’ are often inadequately operationalised as ‘anxiety’, worry about, or ‘fear’ of symptoms. On the other hand, the HBM is considered a weak predictor of behaviour change as it does not include the formation of an intention to change behaviour as a precursor to behavioural change, does not accommodate social and environmental influences or past behaviour, and assumes that human decision-making are rational [84]. In response to each model’s limitations, a combination of the two may determine behaviour likelihood [84, 85] and as shown in this study, their combination provided improved prediction of non-attendance (i.e. prediction of non-attendance improved significantly from 38.8% to 65.0% when combining all significant predictors). This suggests that interventions could be aimed to incorporate various dimensions of both models.

### Limitations

Although these data can be generalizable to other screening programmes with a similar population, such as Mediterranean populations, this study has some limitations. First, a temporal relationship between exposure and outcome cannot be established because the study was cross-sectional thus excluding causal associations. Second, the study’s retrospective design may have had an impact on the recall of events. Third, it was not possible to capture the data of repeat mammograms at another facility as this was not recorded on the screening database at the time of study. Such data would show more accurately women’s adherence to screening guidelines [86] by using multiple points of service. Hence, future research should take into account the type of screening programme and a clear distinction of the type of mammography (screening or diagnostic mammography), since women’s accuracy and consistency in reporting mammography experiences sharply declines with an increased number of lifetime mammograms [87]. Fourth, data collected might be affected by recall or social-desirability response bias i.e. having performed mammography, whether in the organized screening programme or as opportunistic screen due to its well-publicized recommendation by media and clinicians [17], thus amplifying the recall bias effects.

Additional research is required to test the interactions of HBM and CSM components in multivariate models to test threat representations and coping mechanisms. Further research to measure health beliefs and illness perceptions before and after screening could help to clarify the value of HBM and CSM in explaining the beliefs and perceptions of BC risks. Additionally, a longitudinal study design could provide a better understanding of the psychological and emotional pathways and processes involved in how individuals form beliefs and risk perceptions of a particular health threat to better understand the factors underpinning health behaviours and reduce BC risk. Further research is warranted to determine whether uptake to first screening invitation is a significant predictor of subsequent screening in Malta.

## Conclusions

The present study showed that there is high awareness of BC signs and symptoms among Maltese women, but wide variation in knowledge about causes of BC and its related risk factors. Non-attendees were the most unsure of BS recommended practices and had higher emotional barriers. Interventions to increase BC screening uptake in Malta should address health beliefs, in particular perceived barriers such as fear, since these emerged as the strongest predictors of uptake throughout the analyses. However, those interventions that also address illness representations, such as causes, consequences and cyclical timeline of BC, may increase their effectiveness since these were also found to be associated with BS uptake. The CHBMS-MS and IPQ-R variables that contributed most to the regression model were perceived barriers, cues to action and self-efficacy, causes of BC, cancer cyclical timeline, BC consequences and personal control. The findings of this study indicate that it was the combination of both HBM and CSM constructs which provides improved prediction of non-attendance. To our knowledge, this is an innovative finding in BS research. This study provides valuable information to healthcare providers, researchers, screening leads and public health educators as the findings can aid to design culturally sensitive interventions to improve screening behaviours.

## Additional file

Additional file 1: STROBE 2007 (v4) Statement—Checklist of items that should be included in reports of cross-sectional studies (DOCX 20 kb)

## Abbreviations

BC: Breast cancer
CHBMS-MS: Champion’s Health Belief Model Scale for Mammography Screening
CI: Confidence interval
CSM: Common-Sense Model
GP: General practitioner
HBM: Health Belief Model
IPQ-R: Revised Illness Perception Questionnaire
M: mean score
MBSP: Maltese Breast Screening Programme
OR: Odds ratio
SD: Standard deviation
STROBE: Strengthening the Reporting of Observational Studies in Epidemiology
